# Targeting of CDK9 with indirubin 3’-monoxime safely and durably reduces HIV viremia in chronically infected humanized mice

**DOI:** 10.1371/journal.pone.0183425

**Published:** 2017-08-17

**Authors:** Sandra Medina-Moreno, Thomas C. Dowling, Juan C. Zapata, Nhut M. Le, Edward Sausville, Joseph Bryant, Robert R. Redfield, Alonso Heredia

**Affiliations:** 1 Institute of Human Virology, University of Maryland School of Medicine, Baltimore, Maryland, United States of America; 2 Department of Pharmaceutical Sciences, Ferris State University, Grand Rapids, Michigan, United States of America; 3 Marlene and Stewart Greenebaum Cancer Center, University of Maryland School of Medicine, Baltimore, Maryland, United States of America; George Mason University, UNITED STATES

## Abstract

Successful propagation of HIV in the human host requires entry into a permissive cell, reverse transcription of viral RNA, integration into the human genome, transcription of the integrated provirus, and assembly/release of new virus particles. Currently, there are antiretrovirals against each of these viral steps, except for provirus transcription. An inhibitor of HIV transcription could both increase potency of treatment and suppress drug-resistant strains. Cellular cyclin-dependent kinase 9 (CDK9) serves as a cofactor for the HIV Tat protein and is required for effective transcription of the provirus. Previous studies have shown that the CDK9 inhibitor Indirubin 3’-monoxime (IM) inhibits HIV transcription *in vitro* and in short-term *in vivo* studies of HIV acute infection in humanized mice (PBMC-NSG model), suggesting a therapeutic potential. The objective of this study is to evaluate the toxicity, pharmacokinetics and long-term antiviral activity of IM during chronic HIV infection in humanized mice (HSC-NSG model). We show that IM concentrations above EC_50_ values are rapidly achieved and sustained for > 3 h in plasma, and that non-toxic concentrations durably reduce HIV RNA levels. In addition, IM enhanced the antiviral activity of antiretrovirals from the reverse transcriptase, protease and integrase inhibitor classes in *in vitro* infectivity assays. In summary, IM may enhance current antiretroviral treatments and could help achieve a “functional cure” in HIV patients by preventing expression of proviruses.

## Introduction

Cellular human positive transcription elongation factor (P-TEFb), composed of cyclin-dependent kinase 9 (CDK9) and cyclin T1, regulates RNA Polymerase II dependent transcription of cellular and integrated HIV genes [1–6]. CDK9, unlike most other CDKs, controls gene transcription and has little effect on cell cycle regulation [7]. Approaches targeting CDK9 *in vitro* with catalytic inhibitors [8–10], RNAi [11], and direct inhibition using a dominant negative form [12], have all suggested that inhibition of HIV transcription without toxicity might be possible.

Because CDK9 inhibition suppresses transcription of antiapoptotic proteins [13, 14], several CDK9 inhibitors are currently in clinical development for the treatment of cancer [15–24]. However, these inhibitors may have off-target toxicities [18–20, 25–27], suggesting safer CDK9 inhibitors are needed. Indirubin and its derivatives have been used successfully in China for the treatment of chronic myelogenous leukemia [28]. They act by competitively inhibiting ATP binding to the catalytic domain of several CDKs [29]. The indirubin derivative indirubin 3’-monoxime (IM) inhibits CDK9 more potently than other CDKs [30], it is not cytotoxic to primary lymphocytes and macrophages [30, 31], and it is more soluble than indirubin [29]. We [30], and others [31, 32], have previously shown that IM inhibits Tat-mediated elongation of HIV transcripts, and virus replication in primary lymphocytes and macrophages (IM EC_50_ values of 1 and 0.5 μM, respectively). We have also shown that IM suppresses HIV viremia and preserves CD4/CD8 ratios in NSG mice transplanted with human PBMCs (PBMC-NSG mice) [33]. However, these studies could only evaluate the antiviral activity of IM in the short-term (18 days) because of inherent limitations of the PBMC-NSG mouse model, namely, animal deterioration due to graft-versus-host disease (GVHD). In addition, HIV replication in PBMC-NSG mice resembles acute, rather than chronic, infection in humans because depleted lymphocytes are not replenished and HIV viremia cannot be sustained [34, 35].

The potential use of CDK9 inhibitors, such as IM, in HIV patients will likely involve treatment during chronic infection and for prolonged periods of time. In the present study, we report IM toxicity and pharmacokinetics for the first time. We also report the antiviral activity of IM during chronic HIV infection using NSG mice transplanted with human CD34+ cells (HSC-NSG mice), a model that allows continuous production of lymphocytes and supports HIV replication for extended periods of time as in humans [35–40]. Together, the data demonstrate that IM has favorable pharmacokinetics, and that IM can safely and durably reduce viremia in humanized mice with chronic HIV infection, suggesting it could help control HIV in patients.

## Materials and methods

### Ethics statement

All research with human samples and mice was performed in compliance with the institutional guidelines and the US Department of Health and Human Services Guide for the Care and Use of Laboratory Animals. The Committee on Animal Care at the University of Maryland School of Medicine reviewed and approved the described studies. Mice were monitored daily for morbidity and mortality, and euthanized immediately if any of the alternative endpoints was met. The alternative endpoints included a weight loss exceeding 20% as compared to day 0, signs of sluggishness, diarrhea (debilitating or prolonged for 2–3 days), postural hunching, fur ruffling, alopecia (covering at least 25% of body surface area), loss of appetite, GVHD, and ocular trauma. The euthanization method for mice of age 7 days or older was CO_2_ asphyxiation followed by cervival dislocation. For mice younger than 7 days, the euthanization method was decapitation with sharp scissors.

### Toxicity studies

IM (Cayman Chemicals, Ann Arbor, MI) was dissolved in 10% Cremophor EL (Sigma, St. Louis, MO). Adult, female BALB/c mice were treated with IM (2.5, 5, and 20 mg/kg; n = 5 per dose) or its vehicle (10% Cremophor EL; n = 5) daily, via intraperitoneal (i.p.) injections, for 14 days. We used mice from a single sex to reduce potential variability of the data, although gender is not expected to have an effect on the toxicity of IM. On day 14, animals were anesthesized with ketamine-xylazine (100-150mg/kg Ketamine + 10-16mg/kg Xylazine) and, after confirming (via toe pinch) that a deep surgical plane of anesthesia had been reached, blood was collected by cardiac puncture. Blood samples were submitted for measurements of blood chemistry profiles and of blood counts.

### Generation and infection of humanized mice

NSG mice were humanized at the age of 2–3 days, following whole body irradiation (10cGy) and hepatic injection of 1.2x10^5^ human cord blood-derived CD34+ cells (Lonza, Walkersville, MD). At week 12, mice were checked for human cell reconstitution by double staining with FITC-conjugated antihuman CD45 antibody (BD Pharmingen) and APC-conjugated antimouse CD45 antibody (BD Pharmingen). Mice were considered successfully transplanted if they had ≥ 5% of human CD45+ cells in peripheral blood. Successfully transplanted mice were infected, via i.p. injection, with 15,000 TCID_50_ units of the CCR5-tropic HIV reference strain BaL.

### Pharmacokinetic data analysis

Fasting, female NSG mice (n = 16) were injected with 40 mg/kg IM via i.p. Blood samples were obtained pre-dose (30 minutes before compound injection) and at 5, 15, 30, 60, 120, 180, 240 and 300 minutes after injection using a sparse sampling design (n = 2 samples per mouse, n = 4 samples per time point). Separated plasma was further extracted and processed for analysis, and IM concentrations determined using HPLC analysis. In brief, sample processing involved extraction of plasma (30 μL) with 400 μL acetonitrile and 2 μg/mL internal standard (estrone sulfate). The sample was briefly vortexed and centrifuged (12 minutes, 6°C), with transfer of 400 μL of supernatant to a clean test tube. The sample was evaporated to dryness under nitrogen, reconstituted in 50 μL of methanol:water (75:25), and 20 μL was injected onto the column. The concentration of primary analyte (IM) and internal standard (IS) d4-estrone were determined using a Shimadzu Nexera X2 liquid chromatography system coupled with a SPD-M30A UV-VIS diode array detector. The liquid chromatography separation was performed on a Prodigy ODS-3V C18 column, 250 × 4.6 mm, 5 μm. The mobile phase consisted of 35% water and 65% Methanol (Optima LC-MS grade) delivered at a flow rate of 0.85 mL/min. Retention times for IM and IS were 13 and 15 minutes, respectively, with detection at 280 nm. The IM peak identity was confirmed using the complete 240–600 nm absorbance spectra. The standard concentration curve was linear over the range of 0.025 μg/mL– 4.0 μg/mL using regression plots of IM to internal standard peak area ratios versus drug concentrations derived with 1/x2 weighting. The bias of the analytical method did not exceed 20% (judged by percent deviation from nominal value) for at least two of three concentrations from quality control samples, and the intra-assay precision error was <15% (expressed as coefficient of variation). Non-compartmental pharmacokinetic parameters were determined from pooled average data at each time point using Phoenix WinNonlin v6.4 (Certara, St. Louis, MO).

### Quantification of HIV RNA levels and CD4/CD8 ratios in humanized mice

Plasma HIV RNA levels were quantified by an in-house real-time RT PCR using HIV Gag primers SK38/SK39 and SYBR green dyes, as in our previous studies [33, 41]. The assay has a sensitivity of 150 copies HIV RNA/40μL plasma. Percentages of human CD4+ and CD8+ T cells were determined by flow cytometry analysis using fluorochrome labeled antibodies against human CD4 (clone SK3), CD8 (SK1), CD3 (clone SK7), and CD45 (clone 2D1) (BD Biosciences). Data were acquired in a BD FACSCalibur flow cytometer and analyzed using FLowJo software (v. 9.7.7). CD4/CD8 ratios were calculated and plotted using GraphPad Prism 5.0 software.

### Drug susceptibility assays

The ARTs tenofovir, raltegravir and indinavir were obtained from the NIH AIDS Research and Reference Reagent Program (Gaithersburg, MD). Drug susceptibility assays were performed in PBMCs as previously [42, 43]. Briefly, donor PBMCs were activated by culture with phytohemagglutinin (PHA) for 3 days. Activated PBMCs were infected with HIV (M.O.I of 0.001) for 2 hours in the absence of IM or ARTs, washed to remove non-adsorbed virus, and plated in culture medium containing IL-2 and various dilutions of IM and ARTs alone and in combination. On day 3, fresh medium with fresh drug was added to the cultures. On day 7, cultures were evaluated for HIV production by measuring HIV p24 levels in the culture supernatants by ELISA (PerkinElmer Life Sciences, Inc., Boston, Massachusetts). Data were normalized to p24 levels in drug untreated controls (100%) and EC_50_ values determined by fitting the data to sigmoidal dose-response (variable slope) curves using GraphPad Prism 5.0 software.

## Results

### Pharmacokinetics of IM

**Fig 1:**
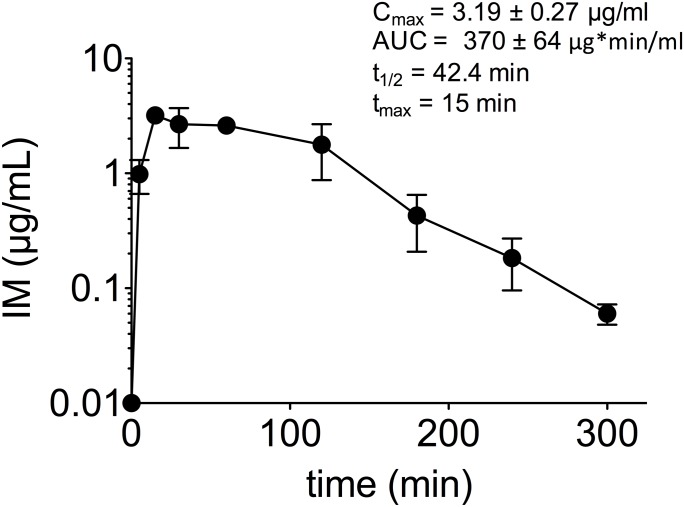
Pharmacokinetics of IM in mice. NSG mice (n = 16) were i.p. injected with 40 mg/kg IM. Blood samples were obtained pre-dose and at 5, 15, 30, 60, 120, 180, 240 and 300 minutes after injection using a sparse sampling design (n = 2 samples per mouse, n = 4 samples per time point). Separated plasma was further extracted and processed for analysis, and IM concentrations were determined using HPLC analysis.

We evaluated the pharmacokinetics of IM in NSG mice following a single i.p. injection of 40 mg/kg, a dose that inhibited plasma HIV RNA levels by 2 log 10 units in our previous study [33]. Data (S1 Table) were plotted on concentration-time curves and pharmacokinetic parameter values determined (Fig 1). The maximum concentration of IM in plasma (C_*max*_) was 3.19 ± 0.27 μg/mL. The time to C_*max*_ (T_*max*_) was 15 min; the AUC was 370 ± 64 μg*min/mL; and the elimination *t*_1/2_ was 42.4 min. These data are consistent with IM antiviral activity because the C_*max*_ values, which correspond to 11.5 ± 0.97 μM, are above the drug EC_50_ values for inhibition of HIV *in vitro* (EC_50_ values of 1 μM in lymphocytes and 0.5 μM in macrophages, [30]). Thus, IM concentrations above HIV EC_50_ values are rapidly achieved and maintained for > 3 h following i.p. injection of 40 mg/kg IM.

### Toxicity of IM

There is currently limited information on IM toxicity [44–46]. In our previous studies in NSG mice, daily IM treatment at doses ranging between 2.5 and 40 mg/kg reduced plasma HIV RNA in a dose dependent manner and increased CD4/CD8 ratios, in the absence of general toxicity [33]. To better evaluate the toxicity of IM we conducted toxicity studies following standard protocols [47]. We used BALB/c mice because, unlike NSG mice, are fully immunocompetent and thus more suitable for assessing hematological toxicities. BALB/c mice were treated with IM (2.5, 5 and 20 mg/kg; n = 5 per dose) or its vehicle (n = 5) daily, via i.p. injections, for 14 days. The highest IM dose evaluated was 20 mg/kg because previous results suggested that the 40 mg/kg dose could interfere with lymphocyte proliferation [33]. Mice were monitored daily for morbidity and mortality. One mouse in the vehicle-treatment control group died in the first week of the experiment, but death did not appear to be related to treatment. The other 19 mice remained healthy and had no evidence of morbidity. Blood tests performed at the end of the study showed values within normal ranges for blood counts and for all chemistry profiles except for creatinine, which were lower in all animals (both IM and vehicle treated) (Table 1). Creatinine levels below reference ranges may be due to sample hemolysis [48, 49]. There were no statistical differences in blood counts or chemistry levels between any of the IM doses and vehicle controls. Thus, these data show that daily IM doses of up to 20 mg/kg do not cause: i) anemia, neutropenia, lymphopenia or thrombocytopenia suggestive of hematologic toxicity; ii) increases on blood urea nitrogen or creatinine suggestive of renal toxicity; or iii) increases on alanine aminotransferase, aspartate aminotransferase, alkaline phosphatase, or bilirubin suggestive of liver toxicity. Together, these data suggest that IM doses of up to 20 mg/kg are not associated with hematologic, renal or liver toxicity.

**Table 1:** Toxicity of IM^a^.

Test	Vehicle (n = 4)	IM (2.5 mg/kg) (n = 5)	IM (5 mg/kg) (n = 5)	IM (20 mg/kg) (n = 5)	Reference values
**Hematology**					
WBC (10^9^/L)	8.9 ± 1.3 (6–12)	6.5 ± 0.6 (5.4–9)	6.8 ± 0.6 (4.9–8.5)	7.1 ± 0.6 (5.5–8.7)	5.7–14.8
ANC (10^9^/L)	0.8 ± 0.2 (0.4–1.3)	1.0 ± 0.1 (0.8–1.5)	1.1 ± 0.1 (0.8–1.4)	1.1 ± 0.1 (0.7–1.4)	0.7–3
ALC (10^9^/L)	7.7 ± 1.4 (5–12)	5.2 ± 0.5 (4.1–7.1)	5.3 ± 0.5 (3.6–6.8)	5.7 ± 0.5 (4.4–7.2)	3.6–11.5
RBC (10^12^/L)	10 ± 0.2 (9.7–10)	9.8 ± 0.1 (9.3–10)	9.6 ± 0.1 (9.3–9.8)	9.4 ± 0.2 (8.7–9.8)	8.2–11.7
**Renal Function or metabolism**					
BUN (mg/dL)	23 ± 2.9 (17–31)	21 ± 1.1 (17–24)	20 ± 0.7 (18–21)	17 ± 0.9 (16–21)	7–31
Creatinine (mg/dL)	0.07 ± 0.02 (0–0.1)	0.16 ± 0.02 (0.1–0.2)	0.14 ± 0.04 (0–0.2)	0.16 ± 0.02 (0.1–0.2)	0.2–0.5
Albumin (g/dL)	2.9 ± 0.07 (2.7–3)	3.0 ± 0.05 (2.8–3.1)	2.8 ± 0.02 (2.8–2.9)	3.0 ± 0.05 (2.8–3.1)	3.0–5.3
Total protein (g/dL)	4.8 ± 0.1 (4.5–5)	4.8 ± 0.05 (4.7–5)	4.8 ± 0.02 (4.7–4.8)	4.8 ± 0.1 (4.6–5.1)	4.9–7.3
Calcium (mg/dL)	8.9 ± 0.6 (7.1–9.6)	7.7 ± 1.8 (0.4–10)	9.5 ± 0.2 (9.1–10)	9.7 ± 0.2 (9.3–10)	8.4–12.7
Phosphate (mg/dL)	9.8 ± 0.8 (7.7–11)	10 ± 0.6 (9.1–12)	9.1 ± 0.7 (7.1–11)	9.1 ± 1.1 (6.3–12)	7.8–13.5
**Liver Function**					
ALT (IU/L)	64 ± 16.6 (34–99)	77 ± 22 (44–161)	131 ± 41 (13–250)	43 ± 8 (23–70)	40–170
AST (IU/L)	327 ± 135 (103–712)	423 ± 84 (283–731)	680±219(216–1451)	324 ± 58 (192–515)	67–381
ALK (IU/L)	154 ± 12 (124–182)	152 ± 35 (13–205)	170 ± 15 (137–220)	177 ± 9 (150–205)	108–367
Total bilirubin (mg/dL)	0.23 ± 0.02 (0.2–0.3)	0.16 ± 0.02 (0.1–0.2)	0.16 ± 0.04(0.1–0.3)	0.24 ± 0.02 (0.2–0.3)	0.2–0.7

^a^ Data are means ± SEM, and range (parenthesis values). BALB/c mice received daily intraperitoneal (ip) injections of vehicle or the indicated doses of IM for 14 days. On day 15, whole blood and serum samples were collected and submitted for hematology and chemistries, respectively.

Abbreviations: WBC, white blood count; ANC, absolute neutrophil count; ALC, absolute lymphocyte count; RBC, red blood count; BUN, blood urea nitrogen; ALT, alanine aminotransferase; AST, aspartate aminotransferase; ALK, alkaline phosphatase.

### Antiviral activity of IM during chronic HIV infection

**Fig 2:**
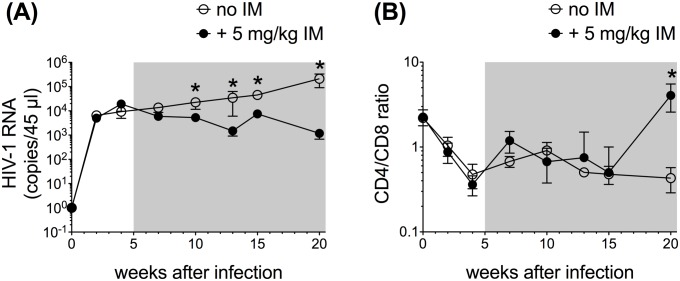
IM reduces HIV viremia in HSC-NSG chronically infected with HIV. Twelve-week old HSC-NSG mice were infected with HIV BaL. Five weeks after infection, treatment was initiated at IM doses of 0 (vehicle alone) and 5 mg/kg/day. Each group had 5 mice. Treatment was continued for 15 weeks. Blood samples collected at the indicated time points were evaluated for plasma HIV RNA levels by quantitative RT-PCR (**A**), and for CD4/CD8 ratios by Flow Cytometry Analysis (**B**). At each time point, data were tested for statistical significant differences by Mann-Whitney U tests (GraphPad Prism Software, La Jolla, CA); P < 0.05 was considered significant. Shaded boxes indicate duration of treatment.

We next evaluated the antiviral activity of IM in HSC-NSG mice chronically infected with HIV. For this, 12-week old HSC-NSG mice were infected with HIV BaL. Five weeks after infection, animals initiated daily i.p. treatment with IM (5 and 20 mg/kg/day) or vehicle control for 15 weeks. Each experimental and control group had 5 mice. Viral replication was monitored by measuring HIV RNA in plasma by RT-PCR. Blood CD4/CD8 ratios were measured by Flow Cytometry. Mice treated with 5 mg/kg dose had modest, but significant, declines in plasma HIV RNA during the 15-week treatment period (Fig 2a). By the end of the study, plasma HIV RNA levels were 2.1 log_10_ units lower in mice treated with 5 mg/kg IM compared to controls. The CD4/CD8 ratios increased to similar levels in IM (5 mg/kg) and control groups for most of the study duration. By the end of the study, however, CD4/CD8 ratios were significantly higher in mice treated with 5 mg/kg IM (Fig 2b). Mice treated with 20 mg/kg IM had progressive losses in human T cells in blood and were euthanized after 5 weeks of treatment. The limited available data from the 20 mg/kg IM dose showed no consistent inhibition of plasma HIV RNA or increases in CD4/CD8 ratios (S1 Fig). Together, these data demonstrate that 5 mg/kg IM reduces HIV viremia and increases CD4/CD8 ratios during chronic HIV infection of humanized mice.

### Antiviral interactions of IM and antiretrovirals

A potential use of CDK9 inhibitor for the treatment of HIV will probably be in combination with ARTs from other classes. We thus evaluated the antiviral interaction between IM and ARTs from the NRTI, integrase and protease inhibitor classes in PBMCs infectivity assays *in vitro*. We used the virus strain HIV-1 NL4-3 to ensure a robust viral replication in the assays. Although HIV-1 NL4-3 differs from HIV-1 BaL in usage of coreceptor for cell entry, it is expected to have similar sensitivity to the tested ARTs. At 1 μM IM the EC_50_ values of tenofovir, raltegravir and indinavir were decreased by 3-, 3.3- and 2.2-fold, respectively (Table 2 and S2 Table). These enhancements of ART potencies by IM are consistent with targeting of different steps of the HIV cycle by IM and the tested ARTs.

**Table 2:** IM enhances inhibition of HIV_NL4-3_ by tenofovir (TDF), raltegravir (RAL) and indinavir (IND) in PBMCs^1^.

Geometric mean ART EC_50_, (95% CI)^2^		
ART	No IM	+ 1μM IM
TDF	0.60 μM (0.28–1.28)	0.19 μM (0.17–0.22)
RAL	6.98 nM (4.93–9.9)	2.12 nM (0.99–4.5)
IND	7.74 nM (4.56–13.14)	3.55 nM (1.25–10)

^1^ PHA-activated donor PBMCs were infected with virus, in the absence of drug, for 3 h. Infected cells were cultured in IL-2 medium containing drugs.

^2^ HIV p24 values on day 7 after infection were normalized to p24 values in the absence of IM. EC_50_ values were determined by variable slope non-linear regression analysis using GraphPad Prism software.

## Discussion

We propose to improve HIV treatment in patients by inhibiting a step in the virus life cycle that is not targeted with current ARTs, namely, transcription of the provirus. Previous *in vitro* work, including ours, has suggested CDK9 as a potential target for inhibition of HIV transcription [8, 9, 11, 12, 30, 31]. Two previous studies, including one from our group, targeted CDK9 in PBMC-NSG mice, but only for short duration (2–3 weeks) and in the setting of acute infection [33, 50], which is relevant for pre- and post-exposure prophylaxis of HIV. Because HIV therapy is life-long and because CDK9 is required for transcription of host genes, there is a need to test the validity of targeting CDK9 *in vivo* under conditions that resemble treatment of chronic HIV infection in humans.

In the present study, we have evaluated for the first time IM pharmacokinetics and toxicities, and IM control of chronic HIV infection (HSC-NSG mice) during 15-week treatment. We show that IM pharmacokinetics are consistent with IM antiviral activity, demonstrating that therapeutic concentrations of IM are rapidly achieved and maintained in plasma for > 3 h. We also show that daily IM doses of up to 20 mg/kg for 14 days have no hematologic, liver or renal toxicity in immunocompetent BALB/c mice. A 15-week IM treatment (5 mg/kg/day) of HIV chronic infection in HSC-NSG mice progressively decreased viral loads, achieving ~ 2 log_10_ unit inhibition by the end of the study. Decreased HIV viral loads were associated with recovery of CD4/CD8 ratios, but this effect was only evident at the latest time point. That CD4/CD8 ratios did not recover until late in the study, when viral suppression was highest, suggests that inhibition of HIV replication by a certain threshold might be necessary to achieve cell recovery in this animal model. A higher IM dose of 20 mg/kg/day in HSC-NSG mice was discontinued after 5 weeks of treatment because human lymphocytes declined in blood. This decline on human lymphocytes by 20 mg/kg/day IM was unexpected because the same dose was not immunosuppressive in BALB/c mice. It is possible that the irradiation step performed in NSG mice prior to injection of human HSC cells may increase the sensitivity of cell replenishment to IM. This is consistent with irradiation induced systemic toxicities in many cell types (including HSC cells) and interference with stromal cell function [51].

The present study evaluating IM in established HIV infection of HSC-NSG mice shows some apparent differences with our previous study evaluating IM in acute HIV infection of PBMC-NSG mice [33]. Whereas IM demonstrates similar magnitude of HIV inhibition (~ 2 log_10_ units) in both acute and chronic infection, the antiviral activity and CD4/CD8 ratio recovery were delayed in the current study of chronic infection compared to the previous study of acute infection [33]. However, a direct comparison of both studies is not possible because the former study used drug-resistant CXCR4-tropic viruses whereas the latter study used wild-type CCR5-tropic HIV. An appropriate comparison of IM activity during acute and chronic infection will require additional animal experiments using same virus strains.

Our study has several limitations. First, the PK studies were conducted at a single dose and over a short period (5 rather than 24 hours), precluding precise estimates on optimal dosing and frequency. Second, toxicity studies conducted in BALB/c mice, which were chosen because of their immunocompetent status, may not be fully relevant to humanized NSG mice and cannot evaluate toxicity on immune cells of human origen. Third, the antiviral activity of IM in mice was evaluated at doses of 5 and 20 mg/kg only. We did not evaluate higher doses (e.g., 40 mg/kg) because previous studies suggested inhibition of human PBMC proliferation in humanized mice [33]. The IM dose of 5 mg/kg IM inhibited HIV viral loads by ~ 2 log_10_ units, whereas the 20 mg/kg dose was discontinued because it inhibited cell proliferation. It is possible that IM doses in the range of 5–20 mg/kg may have even stronger antiviral effects without compromising cell proliferation. Fourth, although IM enhanced the anti-HIV activities of NRTI, integrase and protease inhibitors in tissue culture experiments, the potential clinical relevance of these observations will require assessment in *in vivo* studies. Despite these limitations, the data demonstrate that targeting CDK9 with IM, which interferes with HIV transcription [30–32], reduces HIV viremia during chronic HIV infection of humanized mice. The antiviral IM dose of 5 mg/kg in mice would translate into an equivalent human dose of 0.4 mg/kg after dividing by an interspecies scaling factor of 12.3 [52]. Targeting HIV transcription may complement the antiviral activity of available inhibitors of reverse transcriptase, integrase and protease if, as suggested by *in vitro* experiments, enhanced ART potency is also seen in future animal studies. IM, and other CDK9 inhibitors, may also potentiate inhibition of HIV transcription by Tat inhibitors [53]. Moreover, IM, unlike some ARTs [54], has favorable penetration in brain [44, 55], suggesting it could help control HIV in long-lived brain macrophages and reduce HIV-associated CNS complications [56, 57]. Addition of IM, or other CDK9 inhibitors, to existing ART regimens may also help control replication of emerging viral variants resistant to ARTs (21, 22). Finally, targeting of HIV transcription may also prevent reactivation of HIV in resting CD4+ T cells, helping to achieve a “functional” cure [58].

## Supporting information

S1 Fig Effect of 20 mg/kg IM on HIV replication and CD4/CD8 ratios in HSC-NSG mice. Twelve-week old HSC-NSG mice were infected with HIV BaL. Five weeks after infection, treatment was initiated at IM doses of 0 (vehicle alone) and 20 mg/kg/day. Each group had 5 mice. Treatment was discontinued after 5 weeks of IM treatment. Blood samples collected at the indicated time points were evaluated for plasma HIV RNA levels by quantitative RT-PCR (**A**), and for CD4/CD8 ratios by Flow Cytometry Analysis (**B**). Shaded boxes indicate duration of treatment.
(TIFF)

S1 Table Pharmacokinetics of IM in NSG mice. NSG mice were given a single i.p. injection of 40 mg/kg. Blood samples collected at the indicated time points were processed and subjected to HPLC analysis as described in the Methods section.
(DOCX)

S2 Table Impact of IM on inhibition of HIV-1 NL4-3 by TDF, RAL and IND in PBMCs. PHA-activated donor PBMCs were infected with NL4-3 in the absence of drugs. Infected cells were cultured in IL-2 medium containing various dilutions of TDF, RAL and IND in the absence and presence of IM. Virus production was measured by p24 ELISA on day 7 after infection.
(DOCX)
