# Supplementary figures and images for: Targeting of CDK9 with indirubin 3’-monoxime safely and durably reduces HIV viremia in chronically infected humanized mice
Source: PLoS One. 2017 Aug 17;12(8):e0183425. doi: 10.1371/journal.pone.0183425 (PMC5560554; doi:10.1371/journal.pone.0183425)

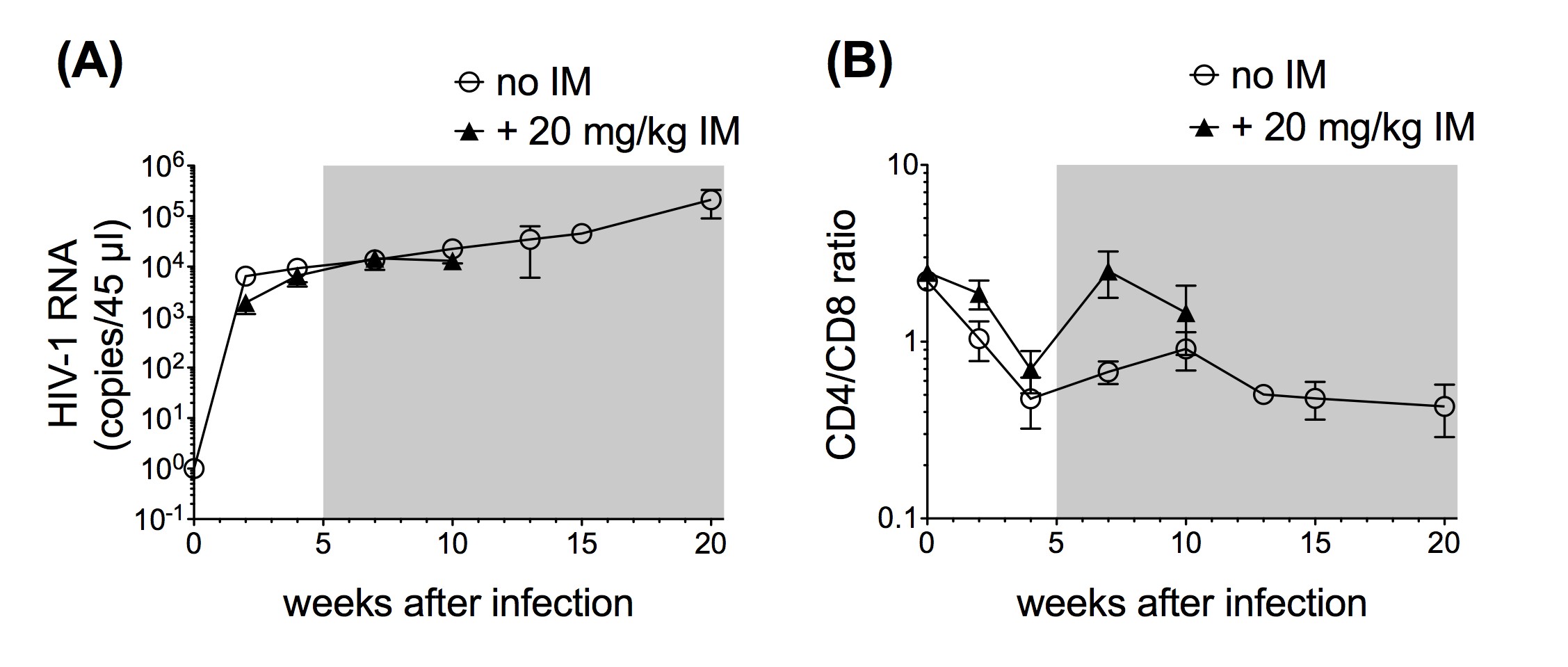

Supplement: S1 Fig — Twelve-week old HSC-NSG mice were infected with HIV BaL. Five weeks after infection, treatment was initiated at IM doses of 0 (vehicle alone) and 20 mg/kg/day. Each group had 5 mice. Treatment was discontinued after 5 weeks of IM treatment. Blood samples collected at the indicated time points were evaluated for plasma HIV RNA levels by quantitative RT-PCR (A), and for CD4/CD8 ratios by Flow Cytometry Analysis (B). Shaded boxes indicate duration of treatment. (TIFF) [file pone.0183425.s001.tiff]
