# Supplementary material for: Targeting of CDK9 with indirubin 3’-monoxime safely and durably reduces HIV viremia in chronically infected humanized mice
Source: PLoS One. 2017 Aug 17;12(8):e0183425. doi: 10.1371/journal.pone.0183425 (PMC5560554; doi:10.1371/journal.pone.0183425)
Supplement: S1 Table — NSG mice were given a single i.p. injection of 40 mg/kg. Blood samples collected at the indicated time points were processed and subjected to HPLC analysis as described in the Methods section. (DOCX) [file pone.0183425.s002.docx]

**Pharmacokinetic Concentrations (IM)^1^**

| **ID #** | **Time (min)** | **Conc (mcg/mL)** |
| --- | --- | --- |
| 1472 | 5 | 1.056 |
| 1473 | 5 | 1.492 |
| 1474 | 5 | 1.336 |
| 1475 | 5 | 0.054 |
| 1468 | 15 | 2.975 |
| 1469 | 15 | 3.996 |
| 1470 | 15 | 2.773 |
| 1471 | 15 | 3.005 |
| 1464 | 30 | 4.918 |
| 1465 | 30 | 2.55 |
| 1466 | 30 | 0.029 |
| 1467 | 30 | 3.217 |
| 1460 | 60 | 2.983 |
| 1461 | 60 | 2.619 |
| 1462 | 60 | 2.828 |
| 1463 | 60 | 2.005 |
| 1472 | 120 | 4.276 |
| 1473 | 120 | 1.171 |
| 1474 | 120 | 1.632 |
| 1475 | 120 | 0.014 |
| 1468 | 180 | 0.38 |
| 1469 | 180 | 1.062 |
| 1470 | 180 | 0.056 |
| 1471 | 180 | 0.217 |
| 1464 | 240 | 0.285 |
| 1465 | 240 | 0.058 |
| 1466 | 240 | 0.014 |
| 1467 | 240 | 0.376 |
| 1460 | 300 | 0.044 |
| 1461 | 300 | 0.079 |
| 1462 | 300 | 0.083 |
| 1463 | 300 | 0.035 |

^1^ Mouse ID # 1466 and 1475 had negligible/undetectable plasma concentrations of IM at each PK draw time point during the study. However, all data was included in the PK analysis.
