# Supplementary material for: Targeting of CDK9 with indirubin 3’-monoxime safely and durably reduces HIV viremia in chronically infected humanized mice
Source: PLoS One. 2017 Aug 17;12(8):e0183425. doi: 10.1371/journal.pone.0183425 (PMC5560554; doi:10.1371/journal.pone.0183425)
Supplement: S2 Table — PHA-activated donor PBMCs were infected with NL4-3 in the absence of drugs. Infected cells were cultured in IL-2 medium containing various dilutions of TDF, RAL and IND in the absence and presence of IM. Virus production was measured by p24 ELISA on day 7 after infection. (DOCX) [file pone.0183425.s003.docx]

**Antiviral activity of tenofovir (TDF), in the absence and presence of IM, in PBMCs^1^**

|  | **P24 (ng/ml)** | |
| --- | --- | --- |
| **TDF (µM)** | **No IM** | **+ IM** |
| 10 | 0.6 | 0.3 |
| 3 | 4.0 | 0.7 |
| 1 | 17.0 | 1.7 |
| 0.3 | 35.0 | 3.8 |
| 0.1 | 48.0 | 6.6 |
| 0.03 | 61.0 | 9.0 |
| 0.01 | 63.0 | 10.0 |
| 0 | 50.0 | 10.0 |

**Antiviral activity of raltegravir (RAL), in the absence and presence of IM, in PBMCs^1^**

|  | **P24 (ng/ml)** | |
| --- | --- | --- |
| **RAL (nM)** | **No IM** | **+ IM** |
| 45 | 0.6 | 0.01 |
| 15 | 2.8 | 1.10 |
| 5 | 17.0 | 2.30 |
| 1.5 | 26.0 | 5.00 |
| 0.5 | 27.0 | 6.50 |
| 0.15 | 23.0 | 6.00 |
| 0.05 | 27.0 | 9.50 |
| 0 | 24.0 | 8.00 |

**Antiviral activity of indinavir (IND), in the absence and presence of IM, in PBMCs^1^**

|  | **P24 (ng/ml)** | |
| --- | --- | --- |
| **IND (nM)** | **No IM** | **+ IM** |
| 150 | 0.01 | 0.01 |
| 50 | 0.01 | 0.01 |
| 16.6 | 17.00 | 0.70 |
| 5.5 | 37.00 | 2.30 |
| 1.8 | 41.00 | 2.00 |
| 0 | 55.00 | 4.00 |

^1^ Each experiment used different donor PBMCs. P24 levels were measured by ELISA on day 7 after infection.
